# Reproductive performance of pandemic influenza A virus infected sow herds before and after implementation of a vaccine against the influenza A (H1N1)pdm09 virus

**DOI:** 10.1186/s40813-019-0141-x

**Published:** 2020-01-23

**Authors:** Sophie Gumbert, Sebastian Froehlich, Anna Rieger, Julia Stadler, Mathias Ritzmann, Susanne Zoels

**Affiliations:** Clinic for Swine at the Centre for Clinical Veterinary Medicine, LMU Munich, Oberschleißheim, Germany

**Keywords:** Pandemic influenza A virus, Reproductive performance, Return to oestrus rate, Field study, Vaccination

## Abstract

**Background:**

Reproductive failure in sow herds due to infection with influenza A viruses has been described in the literature, but only a few studies have focused on the pathogenesis and the clinical signs of the infection. Case reports indicate an association between infections with influenza A viruses and reduced reproductive performance, although it has been difficult to experimentally reproduce the clinical outcome of poor reproductive performance. The aim of the present longitudinal field study was to compare the reproductive performance parameters before and after the implementation of vaccination against the influenza A (H1N1)pdm09 virus in sow herds infected with pandemic influenza A virus. Therefore, farm-specific data of 137 sow herds in Germany, including 60,153 sows, as well as the clinical presentation of the infection were surveyed via questionnaire. Furthermore, average performance parameters (return to oestrus rate, abortion rate, stillbirth rate, number of piglets born alive per litter, preweaning mortality rate and number of piglets weaned per sow per year) were recorded for 6 months before vaccination and 6 months after completion of primary vaccination.

**Results:**

In 79.8% of the farms, the clinical presentation of the infection was characterised by a reduced reproductive performance. These findings were confirmed by analysis of the performance parameters, which revealed a significant decline in the return to oestrus rate (*p* < 0.001), abortion rate (*p* < 0.001) and preweaning mortality rate (*p* = 0.023) and a significant increase of the number in piglets born alive (*p* = 0.001) and piglets weaned per sow per year (*p* < 0.001) after immunisation. The stillbirth rate did not change significantly.

**Conclusion:**

The present study represents the first attempt to demonstrate the association of influenza A virus infection, vaccination and the alteration in reproductive performance parameters, investigating a large number of cases. The results show that by vaccinating against the influenza A (H1N1)pdm09 virus, an improvement in reproductive performance can be achieved in sow herds infected with pandemic influenza A virus. Additionally, the large number of herds that were affected by poor reproductive performance after infection with the aforementioned virus confirms the assumption of an association between pandemic influenza A virus and reproductive losses.

## Background

In April 2009, the influenza A (H1N1)pdm09 virus was detected in humans and, shortly after, provoked the first pandemic of the twenty-first century [1, 2]. Simultaneous to the global spread in humans, the virus emerged in pig farms across the world [3–6]. The porcine origin of the virus is uncontroversial and underlines the importance of the pig as a host for the virus [7].

Influenza A virus (IAV) is a potential pathogen of zoonotic disease and causes worldwide important economic losses [8, 9]. Pathogenesis studies have shown that in pigs as well as in all other mammals viral infection and replication is limited to the respiratory tract [10]. Independent of the subtype, the disease can emerge both in a subclinical and in an acute way, with varying severity, showing febrile illness together with respiratory disease [4, 8, 11]. An involvement in reproductive disorders, such as return to oestrus, abortion or small litters, is assumed, although a causal link could not be shown in all studies [12, 13]. Numerous case reports as well as a case-control study on a naive Norwegian pig subpopulation describe the emergence of reproductive disorders in context with the infection with influenza A virus [14–17]. In contrast, there are few experimental studies investigating the pathogenesis and the clinical presentation of the reproductive losses [18–20]. The clinical presentation was not reproducible in these studies. Thus, a validation of a causal link between the virus infection and inadequate reproductive performance is still lacking. Subsequent to the emergence of the pandemic influenza A virus, a new vaccine against this subtype was developed. The results of the clinical studies for efficacy and safety were verified under field conditions in 315 farms infected with pandemic influenza A virus. A majority of the sows in the affected farms where IAV was circulating showed remarkably reduced reproductive performance. The present longitudinal field study was conducted to evaluate the effect of pandemic IAV on the reproductive parameters and vaccination of animals in conventional sow herds.

## Results

### Clinical signs

Clinical signs prior to vaccination were recorded in 129 farms (Additional file 1, Table S1). Due to information bias, the clinical signs could not be evaluated in eight farms. Reduced reproductive performance was observed in 79.8% (*n* = 103/129) of the farms. The clinical presentation was characterised by fever and respiratory disease (cough) in 62.8% (*n* = 81/129) and 61.2% (*n* = 79/129) of the farms, respectively. Dyspnoea was apparent in 17.1% (*n* = 22/129), reduced feed intake in 39.5% (*n* = 51/129) and apathy in 14.7% (*n* = 19/129) of the farms.

### Reproductive performance

Analysis of the return to oestrus rate revealed, on average, a significant (*p* < 0.001) decline of 3.34% after immunisation (Table 1).

**Table 1:** Reproductive performance data prior to and after implementation of vaccination

	before vaccination		after vaccination			alteration in the farms			
parameter	mean	median	mean	median	*p*-value^a^	decrease (%)	stagnation (%)	increase (%)	
(*n* = number of farms)	(SD)	(Q_25_; Q_75_)	(SD)	(Q_25_; Q_75_)		(n)	(n)	(n)	*p*-value^b^
return to oestrus rate (%)	13.52	12^a^	10.18	9.9^a^	< 0.001	74.8	5.3	19.8	
(*n* = 131)	(6.65)	(8.8; 18)	(4.61)	(7; 12)		(98)	(7)	(26)	< 0.001
abortion rate (%)	2.31	1.45^a^	1.42	1^a^	< 0.001	57	21.5	21.5	
(*n* = 93)	(2.52)	(0.8; 3.0)	(1.67)	(0.4; 2.1)		(53)	(20)	(20)	< 0.001
stillbirth rate (%)	7.79	7.8^a^	7.95	8.2^a^	> 0.05	40	8.0	52	
(*n* = 50)	(3.75)	(5.3; 9.8)	(3.44)	(6.8; 9.9)		(20)	(4)	(26)	0.376
piglets born alive/litter (n)	13.24^a^	13.2	13.56^a^	13.5	0.001	25.9	3.7	70.4	
(*n* = 54)	(1.12)	(12.5; 13.6)	(1.17)	(12.8; 14.2)		(14)	(2)	(38)	0.001
preweaning mortality (%)	14.34	14.7^a^	13.59	13.7^a^	0.023	49.6	16	34.4	
(*n* = 125)	(3.5)	(12.5; 16)	(4.0)	(11.6; 16)		(62)	(20)	(43)	0.08
piglets weaned/sow/year (n)	26.06^a^	26.2	27.39^a^	27.2	< 0.001	18.1	4.8	77.1	
(*n* = 105)	(3.03)	(23.8; 28.5)	(3.15)	(25.5; 29.9)		(19)	(5)	(81)	< 0.001

^a^comparison of the parameters before and after immunisation, t-test or Wilcoxon signed-rank test, respectively

^b^stagnation was not included in the chi-squared test

In 74.8% of the farms (*n* = 98/131) a significant reduction (*p* < 0.001) in the return to oestrus rate was observed after implementation of vaccination (Table 1). In those farms, the mean return to oestrus rate was significantly (*p* < 0.001) reduced by 5.1% (± 4.66). Overall, in 3 farms (2.2%) none of the assessed reproductive parameters improved. The number of farms varies for each parameter because valid data were not available for each assessed reproductive parameter from all farms (Table 1).

The farms were categorised according to the return to oestrus rate before the immunisation. The results of the subsequent analysis show that a high return to oestrus rate before vaccination is associated with a greater decrease in the return to oestrus rate after vaccination. Between the categories significant deviations (*p* < 0.001) in the decrease in the return to oestrus rate were observed (Table 2).

**Table 2:** Categorisation of farms prior to vaccination and respective means of return to oestrus rate after vaccination

category (range of return to oestrus rate)	n	decrease of the return to oestrus rate on n farms (%)	mean decrease of the return to oestrus rate by X% (SD)
1 (< 10%)	43	25 (58.1%)	1.56 (1.43)^a^
2 (≥ 10% - < 20%)	66	52 (78.8%)	4.49 (2.87)^b^
3 (≥ 20%)	22	21 (95.5%)	10.97 (5.4)^c^

*n* = number of farms X = average decrease in the return to oestrus rate in the respective category after immunisation ^abc^ groups with different superscripts had significantly different results(*p* < 0.05)

After immunisation the abortion rate decreased significantly (*p* < 0.001) by an average of 1.8% (± 2.24) in 57% of the farms (Table 1). The number of piglets born alive increased significantly (*p* = 0.001) in 70.4% of the farms (Table 1) by an average of 0.6 (± 0.5) piglets. Analysis of the preweaning mortality rate resulted in a significant average reduction (*p* = 0.023) of 2.29% (± 1.9) in 49.6% of the farms (Table 1). In 34.4% of the farms, it increased by an average of 1.7% (± 1.4) and in 16% of the farms (*n* = 20/125), it remained unaltered. Concurrently, an increase of an average of 1.98 piglets (± 1.82) weaned per sow per year was observed in 77.1% of the farms. However, in 18.1% of the farms (*n* = 19/105), the number of piglets weaned per sow per year decreased by 1.08 piglets.

According to an ANOVA, there was no influence of the month of vaccination on the reproductive performance parameters, except for the month of August on the preweaning mortality (Additional file 1, Table S3).

Linear regression revealed no association between herd size and reproductive performance parameters (Additional file 1, Table S4 and Table S5). Additionally, no association between pre-vaccination of farms against other IAV subtypes and the return to oestrus rate, the abortion rate, the stillbirth rate, the number of piglets born alive/litter or the preweaning mortality rate was observed (Additional file 1, Table S6). However, the number of piglets weaned/sow/year observed after the implementation of vaccination against pandemic IAV was significantly (*p* = 0.016) higher in farms that were already pre-vaccinating against other influenza subtypes than in farms that were not doing so (Additional file 1, Table S6).

## Discussion

Pandemic influenza can induce respiratory disease, such as coughing and dyspnea, as well as elevated body temperature, anorexia and apathy. The results of the survey concerning the clinical signs of the infection with pandemic IAV coincide with the observations of numerous experimental studies and case reports [4, 11, 21]. Interestingly, in the present study, reproductive disorders were present in nearly 80% of the assessed farms. A connection between reduced reproductive performance and infection with IAV is described in the literature, but data confirming a causal link is lacking. Furthermore, the pathogenesis of reproductive disorders subsequent to infection with IAV is not definitively determined. Alterations of the maternal immune system during pregnancy can result in an increased likelihood of influenza virus infection [22]. The few studies examining the effect of infection with IAV during pregnancy were conducted mainly with rodent models [22, 23]. Thus, interpretation of these studies is hampered by histological dissimilarities of the rodent placenta and the of swine placenta [24]. It is assumed that reproductive disorders due to influenza virus infections are indirectly caused by systemic consequences of the infection [25], particularly by fever and immunological reactions leading to hormonal imbalances [26, 27]. An increase in proinflammatory gene expression after infection with influenza virus results in the release of inflammatory cytokines [28, 29]. The pathological consequence of the infection may be reduced progesterone synthesis in the corpus luteum in pregnant animals [23]. Consequently, a low progesterone level induces luteolysis and termination of pregnancy [30].

The vast majority of the sow herds in the present study showed reproductive disorders. After confirmation of an infection with pandemic IAV, the herds were immunised against the pandemic influenza A (H1N1)pdm09 virus. Immunisation against IAV can reduce clinical disease, virus shedding and transmission in infected animals [31, 32]. It was shown previously that by immunisation with the vaccine used in the current study, the viral lung load, virus shedding and clinical parameters such as dyspnoea and elevated body temperature can be reduced in animals infected with pandemic influenza A (H1N1) virus [33, 34]. Reduction in viral lung load and viral shedding after immunisation correlates with reduced proinflammatory cytokine secretion and therefore with the extent of the disease [35].

In the current study, the analysis of the performance parameters revealed significant alterations in the sow herds for the time period after immunisation compared to those for the time period before vaccination. The results for the return to oestrus rate as well as the abortion rate showed, on average, a significant decline after immunisation. Classification of the herds into categories based on the average return to oestrus rate during the time period before vaccination revealed significant differences between the groups. The percentage of returning sows and the ratio of reduction in the return to oestrus rate after vaccination differed significantly. The return to oestrus rate was significantly more reduced after vaccination in sow herds with a high return to oestrus rate before vaccination than in sow herds with a low return to oestrus rate.

It might be hypothesised that the severe clinical outcome of IAV infection in herds might be caused by an additive effect of non-infectious factors as well as coinfections stimulating the immune system in herds with a high return to oestrus rate. It has been shown in experimental studies that a significantly stronger inflammatory response is induced when IAV infection is accompanied by coinfections [36]. Additionally, it has been proven that non-infectious factors such as poor hygienic conditions can stimulate the immune system [37, 38]. This finding could be another explanation of why under experimental conditions, usually with high hygienic standards and under the absence of coinfectious agents, the clinical course of reproductive losses cannot be consistently reproduced [19, 20].

Thus, vaccination in herds with a severe clinical outcome might lead to a more intense reduction in the return to oestrus rate than in herds with mild clinical outcomes. This hypothesis could not be proven by our observation, as it was beyond the scope of the study to perform a detailed assessment of internal biosecurity on the farms or to investigate coinfections other than those known at the beginning of the study. Nevertheless, the varying pathogenicity of different influenza virus strains must also be considered in herds with varying severity in their clinical course [11].

In contrast to the literature, in which an increased occurrence of stillbirths after infection with IAV is described in case reports and experimental studies [15, 18, 39, 40], the stillbirth rate was not altered significantly in the current study. The literature shows that over 70% of stillborn piglets die during parturition caused by non-infectious reasons such as asphyxia or dystocia [41], and only 30% can be attributed to infectious agents. Usually, there are also only a few litters involved [15, 18]; thus, the cases where this pathogenesis could have been seen in the current study are probably overlaid by the usual losses. Furthermore, as the numbers of farms with an increasing and decreasing stillbirth rate were not significantly different, a tendency for improvement was not seen.

Interestingly, this report is the first study describing a significant improvement in the parameters number of piglets born alive per litter, preweaning mortality and piglets weaned per sow and year after immunisation against IAV. On average, these reproductive parameters reached the respective herd-specific benchmark range. The difference in the number of herds with an increasing or a decreasing preweaning mortality rate tended to be significant (*p* = 0.08); for the other parameters, analyses revealed significant differences.

Overall, there was a significant improvement in the reproductive parameters. With the exception of three farms, improvement of at least one reproductive parameter was observed after vaccination. However, the alteration of each parameter differed between farms. These individual levels of influenza affectedness of the single parameter can be explained by various assumptions. There may be different IAV infection time points affecting not all sows in all different gestational states, resulting in different clinical outcomes apparent in the reproductive parameters. Perhaps in the three farms with no improvement in reproductive performance after vaccination IAV infection either remained subclinical or manifested in respiratory disease rather than in reproductive disorders. Furthermore, as it is described that the infection dynamic of IAV can vary from endemic to short epidemic patterns [42], it could be assumed that the assessed time periods did not include the time of clinical disease in the mentioned farms. Additionally, the presence of stress or other immunosuppressive agents might have adversely affected the onset of immunity. After all, vaccine management and handling procedures were not monitored, so lack of compliance might be a possible cause as well. The analysis of the effect of pre-vaccination of some herds against other influenza subtypes revealed that the number of piglets weaned per sow and year improved significantly (*p* = 0.016) more in herds pre-vaccinating sows than in herds that only vaccinated against the pandemic IAV. Repeated vaccination of pigs with antigens of different IAV strains can induce slight cross-reactions in neutralisation tests with pandemic influenza viruses [43]; however, cross-protective immunity cannot be achieved [44]. The parameter piglets weaned per sow and year is only indirectly affected by the vaccine through improved colostrum quality and quantity transferred from healthier sows and a reduction in virus shedding in the farrowing unit. Thus, the results of the current study may indicate improved conditions for piglet health in the farrowing unit when sows are pre-vaccinated against other influenza strains.

Since the current study is a field study, the conditions of every farm are variable and subject to bias. This condition implies that the included farms varied in health status, genetic origin of the animals, management and geographical location. Hence, only the change in parameters within each sow herd was analysed so that a major part of the factors influencing reproductive performance [45–47] remained unaltered during the observation period. Furthermore, analyses revealed that there was no association between herd size and reproductive performance in the current study.

However, to reduce bias, farms with known coinfections, change in vaccination scheme or any other factor that could influence reproduction were excluded from the study. Except for the start of vaccination against pandemic influenza A farms were not allowed to implement any changes in management. Apart from farm-specific features, a common concern is the variability in reproduction and infection rate caused by seasonality [48, 49]. In the current study, infection took place throughout the year (Additional file 1, Table S2), which is in accordance with results from current studies [12, 50]. In addition, no seasonal influence on reproductive parameters was observed. An association between the month and reproduction was not measurable in the statistical analysis (Additional file 1, Table S3), with the exception of the parameter preweaning mortality in the month of August (*p* = 0.037). However, because the model is based on few case numbers, this sole value should not be overrated.

The study has some potential limitations because potential infectious confounders were not evaluated. To verify the causality of the present findings experimental studies need to be conducted under controlled conditions, which was beyond the scope of the current study. However, given that until now, it was not possible to experimentally reproduce the pathogenesis of reproductive disorders due to influenza A virus infection [19, 20], the present results support the common assumption of an association. The findings of the current field study are substantiated through the combination of the large number of cases and the different assessed parameters predominantly showing the same results.

## Conclusion

The results of the current field study evaluating the reproductive performance of 137 sow herds provide data supporting the often described but seldomly scientifically verified causal link between infections with IAV and reduced reproductive performance. An improvement in reproductive performance by vaccination the against influenza A (H1N1)pdm09 virus in sow herds infected with the respective virus could be achieved. The large number of herds (*n* = 137) and animals (*n* = 60,153) included show the importance of influenza virus infection for conventional farms.

## Methods

The aim of the present longitudinal field study was to evaluate reproductive performance parameters after the implementation of a vaccine against the influenza A (H1N1)pdm09 virus in sow herds infected with pandemic influenza A virus.

The study is based on data from commercial swine breeding herds in Germany. A total of 315 sow herds infected with pandemic influenza A virus were included. Infection with the pandemic influenza A virus was verified by laboratory investigations. In 43 farms of the finally assessed 137 farms (see below), the infection with the aforementioned subtype was confirmed by detection of pandemic IAV-RNA by polymerase chain reaction (PCR). Due to the short period of virus shedding after infection [51], farms (94 farms) with the presence of antibodies against pandemic IAV detected by haemagglutination inhibition (HI) were also included in the study. The HI test was performed to detect antibodies against the pandemic IAV and the major European subtypes H1N1, H1N2 and H3N2. In the case of the presence of antibodies against different subtypes, the respective farm was only included if the titre against H1pdmN1 was twofold higher than that against the other subtypes. However, due to the occurrence of possible cross-reactivity in the HI test with other influenza subtypes [52], farms with questionable serologic results were excluded.

The 315 farms were part of an extended field study that was conducted to achieve market authorisation for the vaccine against the pandemic influenza A (H1N1)pdm09 virus (RESPIPORC FLUpanH1N1, IDT Biologika GmbH) by the European Marketing Agency. The field study was conducted in accordance with paragraph 11 section 1 of the German animal health law (formerly paragraph 17 c of the law on epizootic diseases). For the permission of such preregistration trials, either the detection of the virus by PCR or a positive HI test is a mandatory requirement.

In this field trial, primary vaccination was performed on all farms as a classical mass vaccination of all sows, consisting of two vaccinations at an interval of 3 weeks. The time period between the first occurrence of clinical signs related to influenza infection in the herd and the diagnosis and administrative approval differed between herds from 3 to 12 weeks.

The first step of the present study was to collect farm-specific data as well as information on the clinical presentation of the infection on the 315 farms via a standardised questionnaire (Additional file 2). Based on the results of the questionnaire, data from only 137 farms were statistically analysed. The reasons for exclusion were factors that could bias the data, such as restocking of the sow herds, depopulation and repopulation, lack of vaccine compliance or change in the internal biosecurity as well as external biosecurity measures. Additionally, farms with known coinfections with other pathogens, including influenza viruses other than the pandemic subtype, were excluded from the study. However, no further diagnostic investigations assessing coinfections were performed during this study.

To evaluate reproductive performance, the production parameters of the sow herds were recorded for 6 months before implementation of the vaccine and 6 months after completion of primary vaccination. Because the primary basic vaccination consists of two vaccinations at an interval of 3 weeks, this period of 3 weeks was not included in the data records. In detail, the return to oestrus rate, abortion rate, stillbirth rate, piglets born alive per litter, preweaning mortality rate and number of piglets weaned per sow per year were assessed based on routinely recorded production data that were obtained monthly whenever possible. However, the information value of the monthly data was limited due to differing batch farrowing intervals that would distort the monthly data. Therefore, in the current analyses, the data were reduced for every farm to two data points summarising the two time periods (6 months before implementation of the vaccine and 6 months after completion of primary vaccination) to ensure comparability between farms. The farms were categorised by means of the return to oestrus rate before vaccination. The categories were chosen by the following benchmarks: return to oestrus rate < 10% (category 1), return to oestrus rate ≥ 10% and < 20% (category 2) and return to oestrus rate ≥ 20% (category 3), based on a literature review and industry reports characterising average German performance data [53–55]. Subsequently, the alteration in the return to oestrus rate after the implementation of the vaccine was assessed for the different categories.

**Fig. 1:**
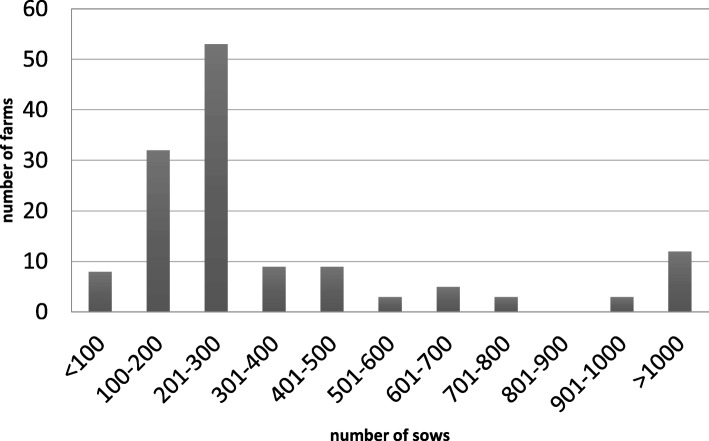
Herd sizes of the included farms

The 137 included farms comprised 104 piglet producing farms, 27 farrow-to-finish herds and 6 multiplier herds. In total, 111 farms were already vaccinating against other subtypes of IAV prior to the implementation of the pandemic IAV vaccine. The herd size ranged from 38 to 5600 sows, with a mean of 448 sows (Fig. 1).

In total, 60,153 sows were included. The locations of the included farms reflect the geographical distribution of the pig population in the German federal states (Table 3).

**Table 3:** Geographical distribution of sow herds included in the study and population in German federal states

State	herds included		pig population * (%)
	number (n)	percentage (%)	
Lower Saxony	52	38.0	31.7
North Rhine-Westphalia	48	35.0	26.3
Bavaria	15	10.9	12.1
Baden-Wuerttemberg	9	6.6	6.4
Schleswig-Holstein	4	2.9	5.3
Saxony-Anhalt	3	2.2	4.3
Mecklenburg-West Pomerania	0	0	3.1
Brandenburg	2	1.5	2.8
Thuringia	1	0.7	2.8
Saxony	1	0.7	2.5
Hesse	1	0.7	2.0
Rhineland-Palatinate	1	0.7	0.6
Saarland	0	0	0.01

* Destatis [56]

### Statistical analysis

The collected data from the questionnaires were summarised in a database using Microsoft Excel® 2010 (Fa. Microsoft, Redmond, USA) and analysed statistically in IBM SPSS® Statistics Version 23.0 (Fa. IBM Corp. Armonk, USA). For descriptive statistics, an exploratory data analysis was carried out. Subsequently, a Wilcoxon signed-rank test or a paired t-test was performed on the paired samples, depending on wether the metric variables were normally distributed. To test whether the numbers of herds with increasing or decreasing reproductive parameters differed, a one-sample chi-square test was performed. For analysis of variance (ANOVA), R software version 3.3.12016 (R Core Team, Vienna, Austria) was used. To test the association between herd size and reproductive performance or vaccination against other IAV subtypes and reproductive performance, a linear regression model was employed. Each farm was considered a statistical unit. The applied level of significance was 5% (*p* < 0.05).

## Supplementary information

**Additional file 1: Table S1.** Clinical signs of the sows of the surveyed farms (*n* = 129).**Table S2.** Year and month of immunisation of each sow herd (*n* = 137). **Table S3.** Analyses of variance (ANOVA) of the reproductive performance parameters influenced by the factor month. **Table S4.** Relationship of the herd size with the initial reproductive performance in a linear regression model. **Table S5.** Relationship of the herd size with the alteration of the reproductive performance in a simple logistic regression**. Table S6.** Relationship of pre-vaccination of the farms against other IAV subtypes and the alteration of each reproductive parameter. Depending of the normality of distribution and equality of variance, the statistic test was chosen.

**Additional file 2.** Observation protocol

## Data Availability

The datasets used and analysed during the current study are not publicly available due to certain restrictions concerning confidentiality but are available from the corresponding author on reasonable request.

## Abbreviations

HI: Haemagglutination inhibition
IAV: Influenza A virus
n: Number
PCR: Polymerase chain reaction
Q_25_: First quartile
Q_75_: Third quartile
SD: Standard derivation

## References

[1] Ginsberg M, Hopkins J, Maroufi A, Dunne G, Sunega DR, Giessick J (2009). Swine influenza A (H1N1) infection in two children. Atlanta: Centers for Disease Control and Prevention.

[2] Cohen J, Enserink M (2009). After delays, WHO agrees: the 2009 pandemic has begun. Science.

[3] Howden KJ, Brockhoff EJ, Caya FD, McLeod LJ, Lavoie M, Ing JD (2009). An investigation into human pandemic influenza virus (H1N1) 2009 on an Alberta swine farm. Can Vet J.

[4] Holyoake P, Kirkland P, Davis R, Arzey K, Watson J, Lunt R (2011). The first identified case of pandemic H1N1 influenza in pigs in Australia. Aust Vet J.

[5] Snoeck CJ, Abiola OJ, Sausy A, Okwen MP, Olubayo AG, Owoade AA (2015). Serological evidence of pandemic (H1N1) 2009 virus in pigs, west and Central Africa. Vet Microbiol.

[6] Mukherjee A, Nayak MK, Dutta S, Panda S, Satpathi BR, Chawla-Sarkar M (2016). Genetic characterization of circulating 2015 a(H1N1)pdm09 influenza viruses from eastern India. PLoS One.

[7] Smith GJD, Vijaykrishna D, Bahl J, Lycett SJ, Worobey M, Pybus OG (2009). Origins and evolutionary genomics of the 2009 swine-origin H1N1 influenza a epidemic. Nature.

[8] Van Reeth K, Brown I, Olsen C. Influenza virus. In: Zimmerman J, Karriker L, Ramirez A, Schwartz K, Stevenson G, editors. Diseases of Swine. Chichester: Wiley-Blackwell; 2012. p. 557–71.

[9] Freidl G, Meijer A, de Bruin E, de Nardi M, Munoz O, Capua I (2014). Influenza at the animal-human interface: a review of the literature for virological evidence of human infection with swine or avian influenza viruses other than a (H5N1). Euro Surveill.

[10] De Vleeschauwer A, Atanasova K, Van Borm S, van den Berg T, Rasmussen TB, Uttenthal A (2009). Comparative pathogenesis of an avian H5N2 and a swine H1N1 influenza virus in pigs. PLoS One.

[11] Brookes SM, Nunez A, Choudhury B, Matrosovich M, Essen SC, Clifford D (2010). Replication, pathogenesis and transmission of pandemic (H1N1) 2009 virus in non-immune pigs. PLoS One.

[12] Harder TC, Beilage EG, Lange E, Meiners C, Dohring S, Pesch S (2013). Expanded cocirculation of stable subtypes, emerging lineages, and new sporadic reassortants of porcine influenza viruses in swine populations in Northwest Germany. J Virol.

[13] Meiners C, Loesken S, Doehring S, Starick E, Pesch S, Maas A (2014). Field study on swine influenza virus (SIV) infection in weaner pigs and sows. Tierärztl Prax Ausg Grosstiere Nutztiere.

[14] Madec F, Kaiser C, Gourreau JM, Martinatbotte F (1989). Pathological consequences of a severe outbreak of swine influenza (H1N1 virus) in the non-immune sow at the beginning of pregnancy, under natural conditions. Comp Immunol Microbiol Infect Dis.

[15] Gourreau JM, Kaiser C, Madec F, Labie J, Vannier P, Aymard M (1985). Passage du virus grippal par la voie transplacentaire chez le porc, dans les conditions naturelles. Annales de l'Institut Pasteur/Virologie.

[16] Karasin AI, Olsen CW, Anderson GA (2000). Genetic characterization of an H1N2 influenza virus isolated from a pig in Indiana. J Clin Microbiol.

[17] Grøntvedt CA, Er C, Gjerset B, Germundsson A, Framstad T, Brun E (2011). Clinical impact of infection with pandemic influenza (H1N1) 2009 virus in naive nucleus and multiplier pig herds in Norway. Influenza Res Treat.

[18] Wesley RD (2004). Exposure of sero-positive gilts to swine influenza virus may cause a few stillbirths per litter. Can J Vet Res.

[19] Kwit K, Pomorska-Mol M, Markowska-Daniel I (2015). Pregnancy outcome and clinical status of gilts following experimental infection by H1N2, H3N2 and H1N1pdm09 influenza a viruses during the last month of gestation. Arch Virol.

[20] Kwit K, Pomorska-Mol M, Markowska-Daniel I (2014). The influence of experimental infection of gilts with swine H1N2 influenza a virus during the second month of gestation on the course of pregnancy, reproduction parameters and clinical status. BMC Vet Res.

[21] Williamson SM, Tucker AW, McCrone IS, Bidewell CA, Brons N, Habernoll H (2012). Descriptive clinical and epidemiological characteristics of influenza a H1N1 2009 virus infections in pigs in England. Vet Rec.

[22] Littauer EQ, Esser ES, Antao OQ, Vassilieva EV, Compans RW, Skountzou I (2017). H1N1 influenza virus infection results in adverse pregnancy outcomes by disrupting tissue-specific hormonal regulation. PLoS Pathog.

[23] Kim JC, Kim HM, Kang YM, Ku KB, Park EH, Yum J (2014). Severe pathogenesis of influenza B virus in pregnant mice. Virology.

[24] PrabhuDas M, Bonney E, Caron K, Dey S, Erlebacher A, Fazleabas A (2015). Immune mechanisms at the maternal-fetal interface: perspectives and challenges. Nat Immunol.

[25] Christianson WT (1992). Stillbirths, mummies, abortions, and early embryonic death. Vet Clin N Am Food Anim Pract.

[26] Vannier P (1999). Infectious causes of abortion in swine. Reprod Domest Anim.

[27] Yoon KJ, Janke BH, Morilla A, Zimmerman JJ (2008). Swine influenza: etiology, epidemiology, and diagnosis.

[28] Ma W, Belisle SE, Mosier D, Li X, Stigger-Rosser E, Liu Q (2011). 2009 pandemic H1N1 influenza virus causes disease and upregulation of genes related to inflammatory and immune responses, cell death, and lipid metabolism in pigs. J Virol.

[29] Pomorska-Mól M, Kwit K, Markowska-Daniel I, Kowalski C, Pejsak Z (2014). Local and systemic immune response in pigs during subclinical and clinical swine influenza infection. Res Vet Sci.

[30] Diehl JR, Day BN (1974). Effect of prostaglandin F2α on luteal function in swine. J Anim Sci.

[31] Van Reeth K, Labarque G, De Clercq S, Pensaert M (2001). Efficacy of vaccination of pigs with different H1N1 swine influenza viruses using a recent challenge strain and different parameters of protection. Vaccine.

[32] Lee JH, Gramer MR, Joo HS (2007). Efficacy of swine influenza a virus vaccines against an H3N2 virus variant. Can J Vet Res.

[33] Committee for Medicinal Products for Veterinary Use (2017). Respiporc FLUpan H1N1: EPAR - Public assessment report. London, UK.

[34] Pesch S, Fachinger V (2018). Efficacy of Respiporc Flupan H1N1 under field conditions.

[35] Van Reeth K, Van Gucht S, Pensaert M (2002). Correlations between lung proinflammatory cytokine levels, virus replication, and disease after swine influenza virus challenge of vaccination-immune pigs. Viral Immunol.

[36] Pomorska-Mol M, Dors A, Kwit K, Czyzewska-Dors E, Pejsak Z (2017). Coinfection modulates inflammatory responses, clinical outcome and pathogen load of H1N1 swine influenza virus and Haemophilus parasuis infections in pigs. BMC Vet Res.

[37] Le Floc'h N, Jondreville C, Matte JJ, Seve B (2006). Importance of sanitary environment for growth performance and plasma nutrient homeostasis during the post-weaning period in piglets. Arch Anim Nutr.

[38] Pastorelli H, Le Floc'h N, Merlot E, Meunier-Salaün MC, van Milgen J, Montagne L (2012). Sanitary housing conditions modify the performance and behavioural response of weaned pigs to feed- and housing-related stressors. Animal.

[39] Woods G, Mansfield M (1974). Transplacental migration of swine influenza virus in gilts exposed experimentally. Res Commun Chem Pathol Pharmacol.

[40] Wallace G, Elm J (1979). Transplacental transmission and neonatal infection with swine influenza virus (Hsw1N1) in swine. Am J Vet Res.

[41] Cutler R, Fahy V, Cronin G, Spicer E. Preweaning mortality. In: Straw B, Zimmerman J, D’Allaire S, Taylor D, editors. Diseases of Swine. Ames: Blackwell Publishing; 2006. p. 993–1009.

[42] Simon-Grifé M, Martín-Valls GE, Vilar MJ, Busquets N, Mora-Salvatierra M, Bestebroer TM (2012). Swine influenza virus infection dynamics in two pig farms; results of a longitudinal assessment. Vet Res.

[43] Dürrwald R, Krumbholz A, Baumgarte S, Schlegel M, Vahlenkamp TW, Selbitz HJ (2010). Swine influenza a vaccines, pandemic (H1N1) 2009 virus, and cross-reactivity. Emerg Infect Dis.

[44] Everett HE, Aramouni M, Coward V, Ramsay A, Kelly M, Morgan S (2019). Vaccine-mediated protection of pigs against infection with pandemic H1N1 2009 swine influenza a virus requires a close antigenic match between the vaccine antigen and challenge virus. Vaccine.

[45] Barnett J, Hemsworth P (1991). The effects of individual and group housing on sexual behaviour and pregnancy in pigs. Anim Reprod Sci.

[46] Kirkwood RN, Althouse GC, Yaeger MJ, Carr J, Almond GW. Diseases of the reproductive system. In: Zimmerman JJ, Karriker LA, Ramirez A, Schwartz KJ, Stevenson GW, editors. Diseases of Swine. Chichester: Wiley-Blackwell; 2012. p. 329–47.

[47] Hazeleger W, Soede N, Kemp B (2005). The effect of feeding strategy during the pre-follicular phase on subsequent follicular development in the pig. Domest Anim Endocrinol.

[48] Auvigne V, Leneveu P, Jehannin C, Peltoniemi O, Sallé E (2010). Seasonal infertility in sows: a five year field study to analyze the relative roles of heat stress and photoperiod. Theriogenology.

[49] Wegner K, Lambertz C, Das G, Reiner G, Gauly M (2016). Effects of temperature and temperature-humidity index on the reproductive performance of sows during summer months under a temperate climate. Anim Sci J.

[50] Kyriakis C, Brown I, Foni E, Kuntz-Simon G, Maldonado J, Madec F (2011). Virological surveillance and preliminary antigenic characterization of influenza viruses in pigs in five european countries from 2006 to 2008. Zoonoses Public Health.

[51] Swenson SL, Foni E, Saito T, Brown I, OiE World organisation for animal health (2016). Influenza A virus of swine. Manual of Diagnostic Tests and Vaccines for Terrestrial Animals.

[52] Van Reeth K, Labarque G, Pensaert M (2006). Serological profiles after consecutive experimental infections of pigs with European H1N1, H3N2, and H1N2 swine influenza viruses. Viral Immunol.

[53] Schnurrbusch U, Loeffler K, Strauch D (2006). Physiologie und Pathologie der Fortpflanzung weiblicher Tiere. Schweinekrankheiten.

[54] Hinken R (2018). Ferkelerzeugung - Jahresergebnisse 2017/2018.

[55] Polson DD, Marsh WE, Dial GD (1998). Population-based problem solving in swine herds. J Swine Health Prod.

[56] Destatis (2017). Agrarstrukturerhebung. Fachserie 3 Reihe 213.

