# Supplementary material for: Reproductive performance of pandemic influenza A virus infected sow herds before and after implementation of a vaccine against the influenza A (H1N1)pdm09 virus
Source: Porcine Health Manag. 2020 Jan 23;6:4. doi: 10.1186/s40813-019-0141-x (PMC6977244; doi:10.1186/s40813-019-0141-x)
Supplement: Supplementary file 1 — Additional file 1: Table S1. Clinical signs of the sows of the surveyed farms (n = 129).Table S2. Year and month of immunisation of each sow herd (n = 137). Table S3. Analyses of variance (ANOVA) of the reproductive performance parameters influenced by the factor month. Table S4. Relationship of the herd size with the initial reproductive performance in a linear regression model. Table S5. Relationship of the herd size with the alteration of the reproductive performance in a simple logistic regression. Table S6. Relationship of pre-vaccination of the farms against other IAV subtypes and the alteration of each reproductive parameter. Depending of the normality of distribution and equality of variance, the statistic test was chosen. [file 40813_2019_141_MOESM1_ESM.docx]

Table 1
Clinical signs of the sows of the surveyed farms (n=129).

| **number of farms** | **%** | **reduced reproductive performance** | **apathy** | **reduced feed intake** | **fever** | **dyspnoea** | **coughing** |
| --- | --- | --- | --- | --- | --- | --- | --- |
| 26 | 20,16 | **yes** | no | no | no | no | no |
| 16 | 12,40 | **yes** | no | no | **yes** | no | **yes** |
| 14 | 10,85 | **yes** | no | **yes** | **yes** | no | **yes** |
| 9 | 6,98 | **yes** | no | no | **yes** | no | no |
| 7 | 5,43 | **yes** | **yes** | **yes** | **yes** | **yes** | **yes** |
| 6 | 4,65 | **yes** | no | no | no | no | **yes** |
| 6 | 4,65 | **yes** | no | **yes** | **yes** | no | no |
| 5 | 3,88 | no | no | **yes** | **yes** | no | **yes** |
| 4 | 3,10 | **yes** | no | no | **yes** | **yes** | **yes** |
| 4 | 3,10 | no | no | no | no | no | **yes** |
| 3 | 2,33 | **yes** | no | **yes** | **yes** | **yes** | **yes** |
| 3 | 2,33 | **yes** | **yes** | **yes** | **yes** | no | **yes** |
| 2 | 1,55 | no | no | no | **yes** | no | **yes** |
| 2 | 1,55 | no | no | no | no | no | no |
| 2 | 1,55 | no | no | no | **yes** | **yes** | **yes** |
| 2 | 1,55 | no | no | **yes** | **yes** | no | no |
| 2 | 1,55 | no | no | **yes** | **yes** | **yes** | **yes** |
| 2 | 1,55 | no | **yes** | **yes** | **yes** | **yes** | **yes** |
| 2 | 1,55 | **yes** | no | **yes** | no | no | **yes** |
| 2 | 1,55 | **yes** | **yes** | no | **yes** | no | no |
| 1 | 0,78 | no | no | no | no | **yes** | **yes** |
| 1 | 0,78 | no | no | **yes** | no | no | no |
| 1 | 0,78 | no | no | **yes** | no | no | **yes** |
| 1 | 0,78 | no | **yes** | no | no | no | **yes** |
| 1 | 0,78 | no | **yes** | no | **yes** | no | **yes** |
| 1 | 0,78 | **yes** | no | no | no | **yes** | **yes** |
| 1 | 0,78 | **yes** | no | **yes** | no | no | no |
| 1 | 0,78 | **yes** | **yes** | no | **yes** | no | **yes** |
| 1 | 0,78 | **yes** | **yes** | **yes** | no | no | no |
| 1 | 0,78 | **yes** | **yes** | **yes** | no | no | **yes** |

Due to information bias, for eight farms the clinical signs could not be evaluated.

**Table 2
Year and month of immunisation of each sow herd (n=137).**

|  | **jan.** | **feb.** | **mar.** | **apr.** | **may** | **june** | **july** | **aug.** | **sept.** | **oct.** | **nov.** | **dec.** | **per year** |
| --- | --- | --- | --- | --- | --- | --- | --- | --- | --- | --- | --- | --- | --- |
| **2016** | 2 | 0 | 1 | 0 | 0 | 0 | 0 | 0 | 0 | 0 | 0 | 0 | 3 |
| **2015** | 1 | 0 | 0 | 0 | 0 | 0 | 0 | 0 | 1 | 0 | 0 | 0 | 2 |
| **2014** | 0 | 0 | 1 | 0 | 0 | 0 | 0 | 0 | 0 | 0 | 0 | 2 | 3 |
| **2013** | 5 | 5 | 7 | 7 | 3 | 5 | 5 | 1 | 8 | 6 | 3 | 2 | 57 |
| **2012** | 1 | 0 | 0 | 0 | 0 | 8 | 12 | 14 | 2 | 9 | 11 | 4 | 61 |
| **2011** | 0 | 2 | 1 | 0 | 3 | 1 | 1 | 2 | 0 | 1 | 0 | 0 | 11 |
| **total** | 9 | 7 | 10 | 7 | 6 | 14 | 18 | 17 | 11 | 16 | 14 | 8 | 137 |

**Table 3
Analyses of variance (ANOVA) of the reproductive performance parameters influenced by the factor month.**

| **return to estrus rate (n=131)** | **regression coefficient** | **std. error** | **p-value** |
| --- | --- | --- | --- |
| intercept* | -13.3925 | 11.3471 | 0.242 |
| month 2 vs. month 1† | 1.6586 | 4.5665 | 0.718 |
| month 3 vs. month 1 | 6.0310 | 5.2971 | 0.259 |
| month 4 vs. 1 | 2.3819 | 5.2614 | 0.652 |
| month 5 vs. 1 | 4.5220 | 5.8303 | 0.440 |
| month 6 vs. 1 | 3.8266 | 5.5681 | 0.494 |
| month 7 vs. 1 | 0.1025 | 5.3700 | 0.985 |
| month 8 vs. 1 | 0.2792 | 6.3377 | 0.965 |
| month 9 vs. 1 | -4.8813 | 4.9425 | 0.327 |
| month 10 vs. 1 | -5.7641 | 4.5876 | 0.213 |
| month 11 vs. 1 | 1.2324 | 4.6996 | 0.794 |
| month 12 vs. 1 | 0.4814 | 4.9303 | 0.922 |

* intercept = “no“, month of immunisation january; †month 1 = january, month 2 = february, month 3 = march, monath 4 = april, month 5 = may, month 6 = june, month 7 = july, month 8 = august, month 9 = september, month 10 = october, month 11 = november, month 12 = december;

| **abortion rate (n=93)** | **regression coefficient** | **std. error** | **p-value** |
| --- | --- | --- | --- |
| intercept* | -1.605747 | 2.138555 | 0.457 |
| month 2 vs. month 1† | -0.370732 | 0.831726 | 0.658 |
| month 3 vs. month 1 | 0.771658 | 0.969472 | 0.430 |
| month 4 vs. 1 | 0.137278 | 1.076257 | 0.899 |
| month 5 vs. 1 | 0.060520 | 1.070944 | 0.955 |
| month 6 vs. 1 | 0.394736 | 1.023959 | 0.702 |
| month 7 vs. 1 | 0.617909 | 1.060926 | 0.563 |
| month 8 vs. 1 | 1.676332 | 1.163474 | 0.157 |
| month 9 vs. 1 | -0.004988 | 0.948732 | 0.996 |
| month 10 vs. 1 | 0.545417 | 0.894491 | 0.545 |
| month 11 vs. 1 | -1.461940 | 1.050441 | 0.171 |
| month 12 vs. 1 | -0.628106 | 1.206877 | 0.605 |

| **pigs weaned/sow/year (n=105)** | **regression coefficient** | **std. error** | **p-value** |
| --- | --- | --- | --- |
| intercept* | 3.18234 | 3.45524 | 0.3616 |
| month 2 vs. month 1† | -0.41201 | 1.16927 | 0.7261 |
| month 3 vs. month 1 | -0.95464 | 1.31126 | 0.4701 |
| month 4 vs. 1 | 0.59068 | 1.43761 | 0.6830 |
| month 5 vs. 1 | 0.23700 | 1.36968 | 0.8633 |
| month 6 vs. 1 | -0.42723 | 1.42050 | 0.7649 |
| month 7 vs. 1 | -0.52170 | 1.61317 | 0.7478 |
| month 8 vs. 1 | 0.43273 | 1.49529 | 0.7735 |
| month 9 vs. 1 | 0.03197 | 1.31752 | 0.9807 |
| month 10 vs. 1 | 1.68013 | 1.17822 | 0.1602 |
| month 11 vs. 1 | -0.39393 | 1.21222 | 0.7466 |
| month 12 vs. 1 | 0.31131 | 1.35641 | 0.8194 |

| **preweaning mortality (n=125)** | **regression coefficient** | **std. error** | **p-value** |
| --- | --- | --- | --- |
| intercept* | -3.85627 | 2.78213 | 0.17019 |
| month 2 vs. month 1† | 1.46168 | 1.10819 | 0.19154 |
| month 3 vs. month 1 | 1.49712 | 1.45088 | 0.30574 |
| month 4 vs. 1 | 0.86592 | 1.26096 | 0.49456 |
| month 5 vs. 1 | 3.01668 | 1.59008 | 0.06199 |
| month 6 vs. 1 | 1.73885 | 1.39219 | 0.21588 |
| month 7 vs. 1 | 1.16041 | 1.33382 | 0.38732 |
| month 8 vs. 1 | 3.33226 | 1.56613 | 0.03694 |
| month 9 vs. 1 | 1.30363 | 1.23148 | 0.29348 |
| month 10 vs. 1 | 1.80515 | 1.15855 | 0.12378 |
| month 11 vs. 1 | 1.21617 | 1.17791 | 0.30545 |
| month 12 vs. 1 | 0.48264 | 1.30599 | 0.71284 |

| **stillbirths (n=50)** | **regression coefficient** | **std. error** | **p-value** |
| --- | --- | --- | --- |
| intercept* | 0.9732 | 6.3125 | 0.880 |
| month 2 vs. month 1† | -1.0637 | 2.1657 | 0.633 |
| month 3 vs. month 1 | -1.3044 | 1.9430 | 0.516 |
| month 4 vs. 1 | -1.6944 | 2.4484 | 0.503 |
| month 5 vs. 1 | -0.5443 | 6.3617 | 0.933 |
| month 6 vs. 1 | -2.3775 | 3.5853 | 0.521 |
| month 7 vs. 1 | -0.3594 | 3.2740 | 0.915 |
| month 8 vs. 1 | -1.0428 | 2.7629 | 0.713 |
| month 9 vs. 1 | 2.5287 | 2.1020 | 0.254 |
| month 10 vs. 1 | 0.1705 | 2.0286 | 0.935 |
| month 11 vs. 1 | 0.9580 | 2.4055 | 0.698 |
| month 12 vs. 1 | 2.7838 | 3.2629 | 0.412 |

| **pigs born alive/litter (n=54)** | **regression coefficient** | **std. error** | **p-value** |
| --- | --- | --- | --- |
| intercept* | 1.47496 | 1.22667 | 0.2478 |
| month 2 vs. month 1† | 0.63448 | 0.40993 | 0.1425 |
| month 3 vs. month 1 | -0.28268 | 0.38276 | 0.4716 |
| month 4 vs. 1 | 0.21393 | 0.45574 | 0.6455 |
| month 5 vs. 1 | 0.58132 | 1.04835 | 0.5874 |
| month 6 vs. 1 | -0.76770 | 0.53059 | 0.1685 |
| month 7 vs. 1 | -0.19863 | 0.44705 | 0.6632 |
| month 8 vs. 1 | 0.79637 | 0.55980 | 0.1753 |
| month 9 vs. 1 | -0.25114 | 0.42600 | 0.5643 |
| month 10 vs. 1 | -0.23100 | 0.42800 | 0.5973 |
| month 11 vs. 1 | 0.20440 | 0.42815 | 0.6400 |
| month 12 vs. 1 | 0.38695 | 0.53538 | 0.4809 |

**Table 4**

**Relationship of the herd size with the initial reproductive performance in a linear regression model.**

| **term** | **estimate** | **std. Error** | **t value** | **pr(>\|t)** |
| --- | --- | --- | --- | --- |
| (Intercept) | 195.5898 | 801.3023 | 0.244 | 0.809 |
| return to estrus rate | 0.3922 | 11.2460 | 0.035 | 0.972 |
| abortion rate | 25.9532 | 27.4672 | 0.945 | 0.355 |
| piglets weaned/sow/year | 7.2245 | 36.2195 | 0.199 | 0.844 |
| preweaning mortality rate | -0.4976 | 21.9197 | -0.023 | 0.982 |
| stillbirths | -12.7023 | 17.9942 | -0.706 | 0.488 |
| piglets born alive/litter | 5.6473 | 70.9578 | 0.080 | 0.937 |

**Table 5**

**Relationship of the herd size with the alteration of the reproductive performance in a simple logistic regression.**

| **term** | **estimate** | **std.error** | **statistic** | **p.value** |
| --- | --- | --- | --- | --- |
| (Intercept) | 360.563523 | 66.93779 | 5.3865464 | 0.0000242 |
| change_return to estrus rate | 7.872126 | 14.08221 | 0.5590121 | 0.5820665 |
| change_abortion rate | 11.103598 | 49.18574 | 0.2257483 | 0.8235797 |
| change_piglets weaned/sow/year | 40.944332 | 36.62721 | 1.1178666 | 0.2762498 |
| change_preweaning mortality rate | 1.546377 | 25.16313 | 0.0614541 | 0.9515787 |
| change_stillbirths | -14.041032 | 40.58858 | -0.3459356 | 0.7328328 |
| change_piglets born alive/litter | 57.175258 | 131.62561 | 0.4343779 | 0.6684446 |

**Table 6**

**Relationship of pre-vaccination of the farms against other IAV subtypes and the alteration of each reproductive parameter. Depending of the normality of distribution and equality of variance, the statistic test was chosen:**

Wilcoxon rank sum test with continuity correction

| **term** | **p.value** | **statistic** | **alternative** |
| --- | --- | --- | --- |
| change_return to estrus rate | 0.1673868 | 1051.5 | two sided |
| change_stillbirths | 0.1202216 | 122.5 | two sided |

T-test with equal variance

| **parameter** | **estimate1** | **estimate2** | **statistic** | **p.value** | **conf.low** | **conf.high** |
| --- | --- | --- | --- | --- | --- | --- |
| abortion rate | 0.6507143 | 0.9322785 | -0.4711884 | 0.6386338 | -1.4685473 | 0.9054189 |
| preweaning mortality rate | 0.0545833 | 0.6461765 | -1.0801707 | 0.2821626 | -1.6756149 | 0.4924286 |
| piglets born alive/litter | 0.2690000 | 0.3295455 | -0.2729046 | 0.7860074 | -0.5057315 | 0.3846406 |

T-test with unequal variance

| parameter | t | df | p-value |
| --- | --- | --- | --- |
| piglets weaned/sow/year | -2.5279 | 37.176 | 0.01585 |
