# Supplementary material for: Reproductive performance of pandemic influenza A virus infected sow herds before and after implementation of a vaccine against the influenza A (H1N1)pdm09 virus
Source: Porcine Health Manag. 2020 Jan 23;6:4. doi: 10.1186/s40813-019-0141-x (PMC6977244; doi:10.1186/s40813-019-0141-x)
Supplement: Supplementary file 2 — Additional file 2. Observation protocol [file 40813_2019_141_MOESM2_ESM.docx]

| **Vaccination against pandemic Influenza A Virus:**  **observation protocol** | | | |
| --- | --- | --- | --- |
| Report Nr.: | | | |
| Herd attending veterinarian: | | | |
|  | | | |
| **General information:** | | | |
| Address of the farm | |  | |
|  | |  | |
| Production system | | □ multiplier farm  □ farrow to finish farm  □ piglet producing farm  □ fattening farm  □ other: _____ | |
| Number of sows | |  | |
| Number of gilts | |  | |
| Replacement rate | | _____ sows, every _____ week | |
| Gilt acclimatization management | | □ All-In / All-out □ continous | |
| Duration of gilt acclimatization | |  | |
| All-In / All-out system | | □ farrowing unit  □ nursery unit  □ fattening unit | |
| Batch farrowing interval | |  | |
| Estrus synchronization | | □ yes: □ only in gilts □ in gilts and sows  □ no | |
| Mating management | | □ hormone-controlled ovulation  □ spontaneous | |
| Number of nursery places | |  | |
| Number of fattening places | |  | |
| Stocking density | | □ nursery unit: _____  □ fattening unit: _____ | |
| Clinical signs: | | □ coughing  □ dyspnoea  □ fever  □ reduced feed intake  □ apathy  □ reproductive disorders | |
|  | |  | |
| **Current vaccination scheme:** | | | |
| **Sows** | | **Gilts** | |
| PRRSV |  | PRRSV |  |
| Parvovirus |  | Parvovirus |  |
| Influenzavirus |  | Influenzavirus |  |
| *E. coli/Clostridium spp.* |  | *E. coli/Clostridium spp.* |  |
| Circovirus |  | Circovirus |  |
| others |  | others |  |
| Parasite treatment |  | Parasite treatment |  |
|  |  |  |  |

| **Piglets** |  | |  | |  |
| --- | --- | --- | --- | --- | --- |
| *Mycoplasma hyopneumoniae* |  | |  | |  |
| PRRSV |  | |  | |  |
| Influenzavirus |  | |  | |  |
| Circovirus |  | |  | |  |
| others |  | |  | |  |
| Parasite treatment |  | |  | |  |
|  |  | |  | |  |
| **Reproductive performance:** | | | | | |
| Vaccination dates: _________________  time period 6 months before vaccination: _________________  time period 6 months after primary vaccination of all sows and gilts: _________________ | | | | | |
| **Sows** | | **before vaccination** | | **after vaccination** | |
| Return to estrus rate | |  | |  | |
| Abortion rate | |  | |  | |
| Number of piglets weaned per sow and year | |  | |  | |
|  | |  | |  | |
| **Piglets** | | **before vaccination** | | **after vaccination** | |
| Piglets born alive per litter | |  | |  | |
| Piglets born dead (%) | |  | |  | |
| Preweaning mortality rate | |  | |  | |

**Monthly production records should be added!**
